# Experiences of Patient-Led Chronic Pain Peer Support Groups After Pain Management Programs: A Qualitative Study

**DOI:** 10.1093/pm/pnab189

**Published:** 2021-06-28

**Authors:** Michelle Farr, Heather Brant, Rita Patel, Myles-Jay Linton, Nicholas Ambler, Sareeta Vyas, Hannah Wedge, Sue Watkins, Jeremy Horwood

**Affiliations:** 1 The National Institute for Health Research Applied Research Collaboration West (NIHR ARC West) at University Hospitals Bristol and Weston NHS Foundation Trust, Whitefriars, Lewins Mead, Bristol, UK; 2 Population Health Sciences, Bristol Medical School, University of Bristol, Bristol, UK; 3 Pain Management Service, Southmead Hospital, North Bristol NHS Trust, Bristol, UK; 4 Doctorate in Clinical Psychology, Clinical and Applied Psychology Unit, University of Sheffield, Cathedral Court, Sheffield, UK; 5 Doctorate in Clinical Psychology, School of Psychology, Cardiff University, Cardiff, UK

**Keywords:** Chronic Pain, Peer Support, Pain Management Programs, Self-Management, Social Intervention, Co-Production

## Abstract

**Objective:**

A qualitative study of patients’ experiences and the impacts of peer support groups that patients maintained after UK NHS group pain management programs (PMPs).

**Design:**

Long-term impacts of group PMPs remain unclear, with indications that positive effects can fade. We evaluated a model of continued peer support, co-produced by patients and clinicians, to maintain the therapeutic impact of PMP groups. A protocol was implemented that encouraged patients to continue to meet in their established PMP group for patient-led peer support (without clinical input) after PMPs finished. Peer support aimed to consolidate self-management, and advance social life recovery. We examined the impacts that groups had on attendees, and why some dropped out.

**Methods:**

Semi-structured interviews with 38 patients and 7 clinicians, analyzed thematically.

**Results:**

Friendship bonds and mutual understandings of effective ways of coping with pain encouraged participants to maintain recovery following PMPs. After PMP professional involvement has ended, these meetings enabled patients to develop greater agency from the shared sense of helping bring about new achievements or averting setbacks. Peer support extended the understanding of what is possible when living with pain. However, continuing meetings were not right for all. Reasons for not attending included lack of connection with peers.

**Conclusions:**

Co-produced peer support groups after PMPs can be a low-cost, effective social intervention, providing emotional, practical and social benefits, with improved self-management skills, stronger social connections and some reduced use of health services. Project resources for developing peer support meetings after PMPs are freely available online.

## Introduction

Peer support is a potentially useful way to share knowledge and expertise between people with long term health conditions, yet peer support research within chronic pain is “still in its infancy” [1]. In other long-term physical and mental health conditions, peer support for people has been found to improve quality of life, self-efficacy, empowerment, psycho-social and health outcomes [2–6]. Peer support can take many forms: one-to-one, group, face to face or online [2]. Pain research that examines individual one to one peer support evidences positive effects on patients’ self-efficacy and activation, patients valuing the social connections and the opportunity to give and receive support [7, 8]. Clinicians can be positive about the potential of individual one to one peer support, but identify that time and space are the largest barriers to implementation [1]. Patients’ perspectives on the potential of peer support can be mixed, with some concerns about a negative focus on pain, whilst others highlight the opportunity to share coping strategies [9]. There is less research on patients’ and clinicians’ actual experiences and the impacts of taking part in group peer support that uses both experiential and clinical expertise. Peer support has the potential to contribute to two under-represented research areas in the management of pain. First, it enables more attention to be placed on the expertise of the person who experiences chronic pain [10], so that their first-hand knowledge can inform healthcare provision. Second, group peer support is an intervention that can support the social and emotional elements of pain, that can sometimes be neglected in comparison with bio-medical elements [11, 12] .

Peer support networks, where both professional and experiential knowledge can be shared, are an important aspect of co-production [13]. Co-production means that people who use health services are treated as partners with skills and experiential knowledge that can enable care [14, 15]. Co-production is an important way to bring the experiential knowledge of people living with health conditions to healthcare improvement [13, 14]. Co-production was originally conceptualized in the 1970s in the USA, illustrating how citizens can play an active role in producing public services that are of importance to them [16–18]. Citizens’ contributions can enhance the design and quality of services [19], enabling “the expertise of the person with chronic pain” [10] to inform healthcare interventions. Yet there is limited research that explores how co-production can be applied to pain services.

Meeting the social needs of people with pain is associated with better physical and psychological well-being [20]. However, social interventions for chronic pain are rare [12, 21] and there is a need to understand how healthcare professionals can support these [22, 23]. Pain research has focused on patient-professional relationships [24, 25], with the significance of interpersonal peer-to-peer relationships outside the clinical environment being under-examined [26].

The peer support group intervention analyzed in this article comes from a “real-life” problem, where patients identified a lack of ongoing support at the end of their Pain Management Program (PMP), which was solved with patients’ own initiative by co-producing peer support groups and an associated protocol with clinicians’ support. PMPs are a widely established group-based psychological intervention in pain services. Short term outcomes include improvements in mobility, independence, mood, sleep, activity levels and reductions in analgesia dependence [27–31]. However, the benefits of PMPs may decrease after 6 to 12 months [31, 32]. Additional support is needed to enhance long-term PMP outcomes [33], yet there is limited clinical resource to provide this. No quantitative studies have assessed peer support after PMPs, instead comparing peer support within PMPs to control groups of PMP without peer support or wait-list control, with improvements in patient outcomes reported [34–37]. A qualitative study has evaluated a single patient-led peer support group after standard PMP end, interviewing only those who attended [33, 38]; the group enhanced well-being, self-efficacy and pain management. No other studies have analyzed multiple patient-designed and led peer support groups, that have developed from the initial relationships between patients within PMPs.

This study analyzes to what extent and how patient-initiated and led peer support groups after PMPs may lead to enhanced well-being, self-efficacy and improved pain self-management skills for their participants. The article examines patients’ experiences of peer support groups that were initiated during PMPs and organized by PMP patients, interviewing both group attenders and non-attenders to understand the usefulness and effectiveness of group peer support, alongside reasons why people choose not to participate in peer support.

## Methods

### The Intervention

This study took place within a pain clinic at North Bristol NHS Trust (NBT) that serves both an urban and surrounding rural area. The service includes provision of group pain management programs (PMPs), running 3-hour weekly face-to face session, for between 8–12 weeks. These were accessed via General Practitioner (GP), or specialized treatment service referral. Referral criteria include significant pain-related dysfunction and distress where there is adjudged potential for a more successful adaptation to the pain condition.

In 2012 a group of patients who had taken part in a PMP within the NBT pain management services had challenged PMP clinicians to help them find a way to extend the benefits gained from their PMP. Together they co-produced a method that helps to trigger peer support amongst PMP participants after NHS programs have finished, known locally as “follow-on groups” (FGs). The aim of these peer support FGs was to sustain and build on the positive effects of PMPs for the longer term. This is different from having access to other types of peer support, because “follow-on” peer support groups:

- build on the social bonds and mutual support already formed during a PMP
- use preexisting PMP groups as the basis of a social intervention
- combine both the professional knowledge gained from a PMP with the experiential knowledge of patients.

**Figure 1.**
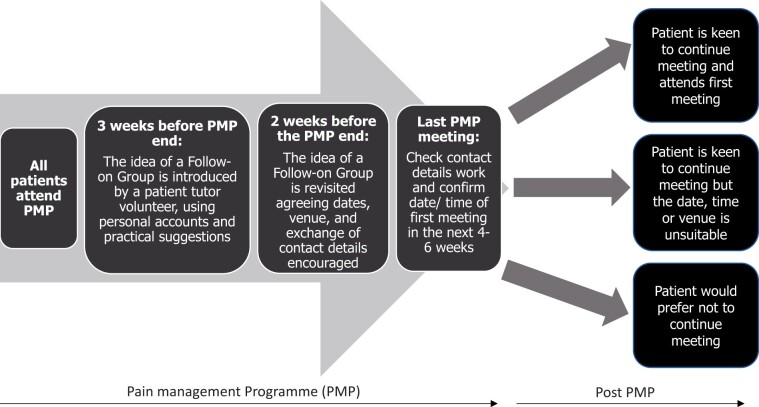
The development of follow-on peer support groups that emerge from the end of PMPs at North Bristol NHS Trust (NBT). PMP = pain management programs.

In usual PMP practice there is a closure phase. In this intervention, three weeks before the end of a course, an experienced patient tutor volunteer (PTV) usually introduces the prospect of building a peer support FG, including personal accounts, information and practical suggestions. PTVs are patients who’ve taken part in a PMP, developed self-management techniques and are trained to co-facilitate PMPs alongside clinicians. They propose a hand-over from the clinical team to the PMP patients, with a framework for running meetings that maintains ongoing peer support without PMP clinicians being present, after the PMP has ended. The PTV and clinician facilitate exploratory discussion to include practicalities and patients taking on organizational roles to make it work. This process unfolds over the final three sessions of the formal PMP treatment phase (see Figure 1). PTVs are tasked to encourage patients within the FGs to continue with goal setting, consolidating what they achieve during PMP and providing mutual support through everyday life and in the face of pain flare-ups. NBT arranges meetings twice a year for previous PMP participants and FG members to discuss how the FGs are progressing, exchange what has been learned, and make contacts between FGs.

### The Research Study

This research was initiated by a group of patients and clinicians who sought support from the National Institute for Health Research Collaboration for Leadership in Applied Health Research and Care (NIHR CLAHRC) West (now the Applied Research Collaboration West) to investigate the experiences of patients who had taken part in FGs after PMPs. The research questions were: i) what types of follow-on groups form (or fold), ii) who is involved or not involved, iii) how are they experienced, and iv) what impacts do they have (or not) and why? A qualitative approach was used to focus upon “people’s subjective experiences within their life context” [10], as the depth and complexity of experiences of pain are not easily understood through quantitative data [11]. The research was reviewed and approved by the NHS Health Research Authority East Midlands—Nottingham 2 Research Ethics Committee (ID: 229806). Data were collected between December 2017 and October 2018.

### Recruitment, Sampling and Data Collection

#### Interviews

A clinician involved in the study (SV) sent out emails to pain clinic colleagues and all PTVs who co-facilitated PMPs, to invite them to take part in the study with an information sheet. Researchers (MF and HB) also visited a PTV meeting at the hospital to introduce the study and give out information leaflets and ask participants to provide contact details if they were interested in taking part in the study.

To recruit PMP patients, information sheets were sent out by a clinician (SV) via post to all patients attending 15 PMPs who had completed a course between 4 and 24 months previously (participants from 12 PMPs responded). PMPs were targeted that had been completed at a minimum of four months previously, so that enough time had elapsed for a follow-on group to potentially form. In addition, to target follow-on groups that been running for a longer period of time, a clinician identified four additional PMPs where an FG had formed and known FG participants were sent invitations to take part (participants from three FGs responded). Researchers aimed to sample between one and three participants from a specific PMP, following maximum variation sampling of different PMPs to analyze alternatives that emerged within different PMPs, so that common patterns across variation could be identified [39]. Verbal (phone interviews) or written consent (face-to-face interviews) was gained, before using an interview topic plan to guide the interviewers (MF, HB) (Supplementary Data). Both face-to-face and phone interviews were offered to provide maximum convenience for research participants. Informal observations of six FGs were also carried out to familiarize and introduce researchers to FG participants. Patients and PTVs received a £10 shopping voucher to thank them for taking part in an interview. Staff received no reimbursement for their time to be interviewed.

### Data Analysis

Interviews were audio recorded, fully transcribed, anonymized, checked for accuracy and imported into NVivo 12 qualitative data software, alongside observation notes. Thematic analysis [40] was conducted by researchers to understand how FGs developed, how PMP dynamics and different contexts affected the development and running of FGs, the activities of FGs, the underlying mechanisms of peer support, patient experiences and the long-term effects of peer support. MF and HB conducted the analysis, first reading the observation and interview transcripts to gain familiarity with the data and initial ideas noted, from which an overarching coding framework was developed, informed by the research questions and interview topic guides. Initial interviews (n = 4) were double-coded to ensure rigor, differences discussed, and the thematic coding framework agreed (MF and HB). The data were scrutinized for differences and similarities within themes across interviewees, and across different PMPs and FGs. The research project team, clinical staff members and a PMP patient involvement group discussed emerging analyses to ensure external validity, rigor, and that the emerging findings were trustworthy and credible.

### Patient Involvement within the Study

As part of the research management team, a patient tutor volunteer (PTV) attended meetings and contributed to initial research design, including how to approach observations of the groups and the best ways of inviting people to participate. Two additional patients commented on patient recruitment information sheets. As the research progressed, the PTV could no longer attend research management meetings due to their ill-health and therefore a separate patient group was set up with other PTVs and patients who had taken part in FGs. Three meetings were held with between five and nine patients and PTVs to feedback on initial findings and discuss methods of disseminating results to help future patients organize FGs. Eight PMP participants and two ARC West public members co-developed clinician and patient information sheets about peer support FGs to share with other PMP facilitators interested in assisting groups to form [41, 42] (Supplementary Data). Ten patients got involved in making a film about their experiences of taking part in peer support FGs [43].

## Results

Interviews were conducted with seven clinicians, ten PTVs, 16 patients who had taken part in FGs, and 12 patients who had not taken part in FGs (from 15 different PMPs; see Table 1 for participant characteristics). To distinguish between a follow-on group (FG), and two friends meeting socially, an FG was defined as at least three PMP participants meeting face-to-face on at least one occasion, after a PMP has finished. Interviews lasted between 18 and 75 minutes (mean 45 minutes); 22 face to face and 23 phone interviews were conducted.

**Table 1.** Interviewee characteristics and sampling

Data Collected	Staff Role (n=)/Clinical Diagnosis (n=) (Some Patients had more than one diagnosis)	Age (years)		Gender	
		Mean (Median)	Range	Women	Men
Staff interviews (n = 7)	Clinical/Health psychologist (4); Physiotherapist (1); Occupational therapist (1); Assistant Psychologist (1).	…	…	5	2
Patient tutor volunteers (n = 10)	Broken back (1); fibromyalgia (5); nerve damage (1); chronic fatigue (1); rheumatoid arthritis (1); arthritis (1); spinal damage from accident (1); not specified (4)	…	…	9	1
Patients attended **at least one** follow-on group (n = 16)	Major spinal surgery (1); adhesive arachnoiditis (1); chronic persistent pain (1); fibromyalgia (5); osteoporosis/arthritis of the spine (2); Carnetts sign (1); trapped nerve (1); complex regional pain syndrome (1); shoulder/back pain with damaged discs (1); chronic fatigue (1); anxiety and mental health problems(1); occipital neuralgia and cervicogenic headaches (1); persistent pain post operation (1). Time living with condition = 2–3 years to 45 years	53 (55)	26–80	13	3
Patients who had **not attended any** follow-on groups (n = 12)	Inflammatory arthritis (1); fibromyalgia (9); psoriatic arthritis (1); spinal accident (1); back pain (1); chronic injury of shoulder and back (1); whiplash from car accident (1); broken neck (1); brain injury (1); ME (2); complex regional pain syndrome (1); sciatica (1); encephalitis from brain cyst (1); hypertension mobility problems (1). Time living with condition = 3 years to 28 years	54 (54)	41–66	12	0

Staff interviewed (labeled S1–S7) had been working in the organization between 2 and 33 years (mean 13 years). Patient tutor volunteers (labeled PTV1–PTV10) came from 10 different PMPs and had completed their PMPs between 6 and 84 months ago (mean 42 months). Of the 10 PTVs interviewed, seven PTVs (PTV 1–2, 5–6, 8–10) had also attended their own FG. Sixteen PMP participants who had attended at least one FG came from nine different PMPs between 5 and 78 months previously (mean 23 months) (labeled P1–P4, P6–P15, P17–P18). Twelve PMP participants who had not attended FGs came from eight different PMPs between 4 and 15 months previously (mean 9 months) (labeled P5, P16, P19–P28). Forty-one interviewees were white British, with four participants reporting different ethnicities (specifics not reported to ensure anonymity). All the participants lived in or the surrounding areas of Bristol including inner city, suburban, and rural areas.

Analysis begins by exploring PMP participants’ social relationships within PMPs, and how these became the bedrock (or not) for the future development of FGs after PMPs. Characteristics and experiences within FGs are explored, before overviewing the impacts of FGs.

### Creation of Follow-On Groups

#### Social Dynamics within PMPs

Social connections and bonding between PMP participants due to shared pain experiences were crucial mechanisms that supported the later development of peer support follow-on groups:

“Things that I thought were crazy in my head, about pain, other people were experiencing it… it made me feel a lot better, and we did gel quite quick. I think because you’re in pain, you gel quicker than somebody who is not in pain” (P2)

Friendship and having a laugh were important to PMP participants, both to those who did and did not take part in subsequent FGs:

“It [PMP] quite quickly became the highlight of my week to go along and see people and have a chat and a laugh with them really” (P25).

Positive stories and the presence of PTVs as role models were highly valued. In a couple of cases PTVs acted as inspirational figures, that catalyzed a transformation in people’s perceptions and behaviors toward their pain, paving the way to a different, more active and fulfilling life:

“What I loved most was her [PTV] story from where she was at the start of her journey to where she’d got to. You think, blimey she’s done it. I can do that. I want that …. She … sold the dream … how much did you want it? I wanted it badly ‘cause like I said I didn’t want to live …. I literally wanted to die. There were times when I planned how I was gonna do it” (P19).

Just over half of the non-attender FG interviewees spoke of more difficult social dynamics within their own PMPs. Negativity within the PMP, an overemphasis on the difficulties of experiencing pain or dominance by people in the PMP group could be problematic:

“Every time the facilitator tried to move on she kept bringing it back to her own condition” (P23).

#### Developing Follow-on Groups After PMPs and Reasons for Not Attending

PMP clinical facilitators and PTVs introduced the idea of follow-on groups usually three sessions before the end of a PMP. This gave the PMP participants enough time to decide if they wanted to take part, exchange contact details, and decide where and when to meet. The original FG protocol suggested that groups were supported in their development by PTVs who were to attend the first three FG meetings and then withdraw. However, PTVs were not always able to attend FG meetings, and only two FGs felt that support from their PTV had been important in helping to establish their FG, with another two having limited support. In three groups, members were already meeting informally and independently before the end of the PMP:

“We kind of became mates and even like well before the thing came to an end, we were, you know, doing things together like going around each other’s houses for a coffee, or going on a night out or stuff like that” (P4).

Connections made through the PMP were crucial to FG development:

“Because in the [PMP] you have to share freely so you’re already set up … it’s confidential so you’re open enough to talk about personal things … you’ve got to know each other … already you’ve broken the ice” (P9).

Where people didn’t meet up, the lack of connection with other PMP participants was a factor: “We weren’t likeminded” (PTV3). Half of FG non-attenders (P5, P16, P19, P20, P23, P25) had not joined FG meetings because of negativity, avoidance of others or the sense that connecting with others may cause decline in their own recovery:

“There were a few people who were always on the negative side, shall we say, and at the end of the course I decided that one of the things I didn’t need was negativity, so I opted not” (P19).

“I can actually feel people’s pain and I experienced quite a lot of it when I was in there, and I ended up looking after a lot of people there” (P16)

Clinical facilitators spoke about PMP group dynamics and how they could affect the subsequent development of FGs:

“There’s definitely been examples of people who want to meet up in groups but then … people irritate them and they’ve often said, ‘Can I go to a different group?’ … sometimes the groups don’t fit together or there’s conflicts .… That’s something we can’t really control” (S6).

Other PMP participants (P24, P28) lost contact with PMP members:

“[The PTV] took everyone’s e-mail address, but I never heard anything, and I’m silly—I should have taken some of their other numbers, ’cause it was a lovely group of people” (P24).

Three interviewees did not attend FGs because of commitments, such as family (P25) and work: “I started going back to work and so, my life’s changed again” (P22, also P19). The logistics of arranging convenient times, dates and travel arrangements could be difficult (P21, P26, P27), complicated by people’s different working patterns (P8, P23, PTV10). Staff and patient interviewees mentioned that language, cultural, and gender barriers (for men, when the majority were women in a group) could be potentially exclusionary for some people.

### Experiences of Follow-on Groups

#### Different Group Characteristics

The groups were diverse in relation to how they communicated, where they met, what they spoke about within their groups and how they functioned (Table 2). There were a broad range of ages, with more women than men meeting, which may reflect the demographics of people attending PMPs. FG members used different mediums to communicate including text (SMS) message, WhatsApp, Facebook, email and telephone calls. The meetings were held in a variety of venues, including cafes, bars and community venues, at varying intervals and were sometimes organized by one person identified as the coordinator. Occasionally, this responsibility could feel like a burden, particularly if the coordinator was unwell. Finding a venue and a time to meet that suited everyone could be challenging and could mean making uncomfortable decisions about who to include. Two interviewees joined a WhatsApp group with several others, but neither spoke of attending a face-to-face FG, which was difficult to organize to fit everyone’s schedules. Some people who had attended groups, were prevented from going regularly due to ill-health:

“I don’t go very often I have to confess because it’s at half past four in the afternoon and normally by that time I’m done in and I can’t drive at that time of the day …. I haven’t been in a while but we text, I get texts and I can still go any time I want” (P18).

**Table 2.** Characteristics of follow-on groups and their members

Group Number	FG Start Date	Number Attending FG (Maximum)	How Often Meeting and Where?	Age Range	Communication Method	Group Duration to Last Contact	Main Purpose of Group as Defined by Members
Group A	2012	9 (1 man, 8 women)	Fortnightly, private community space	40s–80 s	Phone, text (SMS) message	80 months	Social. Goal setting. Pain management techniques. Sharing hobbies and crafts. Exercise, outings together.
Group B	2013	3 women	Monthly, in cafes	50s–70s	Phone, text (SMS) message	60 months	Social. Goal setting. Pain management techniques.
Group C	2016	8 reduced to 6 (1 man, 5 women).	Monthly, in a bar	30s–80 s	Phone	23 months	Social. Pain management techniques. No goal setting.
Group D	2016	6–8 women	6-8 weeks, in cafes	“There’s quite an age gap”	Facebook, text (SMS) message	18 months	Social. Pain management techniques e.g., pacing. No goal setting
Group E	2016	6 women, reduced numbers over time	At each other’s homes, nights out	23–46	Text (SMS) message, Facebook	13 months	Social catch-up
Group F	2017	8 reduced to 6 (1 man, 5 women)	Monthly, private space	40s–70s	Email via group coordinator	15 months	Pain management techniques. Social.
Group G	2017	5–8 (2 men, 6 women), reduced numbers/contact over time	1–2 months, in cafes	20s–67	WhatsApp	12 months	Social. Goal setting. Pain management techniques. Outings
Group H	2017	6 (3 men, 3 women), reduced numbers/contact over time	Monthly, in a bar	30s–60 s	Text (SMS) message	8 months	Social. Pain management techniques.
Group I	2017	6 women, meeting infrequently	2–3 months for a meal	40s and older	Emails and text (SMS) message	7 months	Social. Pain management techniques
Group J	2017	3 (1 man, 2 women), reduced contact over time	Monthly, in cafes	30–41	Facebook, WhatsApp, text	6 months	Social. Pain management techniques
Group K	2017	3 (2 men, 1 woman), only met once	Met once, in a bar	40s–70s	Email	6 months	Pain management techniques

Of those FGs studied, attendance at six groups reduced over time (Table 2: Groups C, F, G, H, J, K), and in Groups B, E, and J those who remained in contact comprised only two or three members. This was due to factors already cited such as loss of contact, fluctuating illnesses, other work or family commitments, and difficulties in arranging times. Calculating time from the final PMP meeting to last research contact with FG members, the groups ranged in duration from 6 to 80 months, with a mean of 22 months (median 13 months) (Table 2).

#### Friendship, Connections and Self-Management

Friendship and the importance of having continued social connections were seen as core to the importance of FGs in people’s lives.

“We understand each other, we don’t judge, and they’re just lovely people and I’ve got two new lovely friends” (P14).

This could combat isolation. Friendships within FGs did not necessarily have a focus on pain. Shared experiences formed a common bond between people, and meant that there was often no need to explain the lived experience of pain:

“There is no pressure to do anything or be anyone other than yourself” (P3).

Eight FG members (P3, P10, P11, P12, P14, P15, P18, P21) spoke about high levels of trust between members, for example sharing things in their groups, that they had not shared with family:

“I’m not really close to my family and I don’t feel that they understand. So, now … I don’t feel on my own or I’m always talking to [P14] and [P8] about it” (P10).

Meeting up together in FGs could be a reason to go out to engage in a social activity, something that could be challenging: “I’m thinking, I’ve done it! I’ve done it! I’m out of my house” (P12). FG meetings could help distract people from their pain:

“I want everyone to have fun because it’s not, it can be pretty dreary sometimes if you’ve got pain all the time. It’s hard work for some people, isn’t it? So, you know, we want to have fun” (P7).

While the original protocol highlighted the importance of FGs focusing on PMP self-management skills, in practice, many saw social connections as the main function of the group. Explicit discussions about self-management techniques varied between groups (see Table 2 for summary):

“I think goal setting has completely gone out the window …. It’s generally just everyday life rather than goal setting, but pacing does come into it” (P3).

“We even sit and discuss, you know, if you go and see your GP, what’s the best way to go about it like they used to in the [PMP] meetings. Make a list. Write it down so as you know exactly what you want to say and just make sure that you’re heard” (P15).

Most staff tended to have a pragmatic approach as to the extent to which FGs focused on self-management skills:

“Ideally if people were able to talk about some of the strategies that we cover in our groups, that would be very pleasing but I don’t know whether that does happen and if it doesn’t happen, you know that’s okay because you know, it’s not, it’s not our group. It’s very much for the participants” (S3).

However, two staff highlighted that if groups “just decide that they want to chat and have a drink together and make it a purely social process that they’ll fall short in what they could potentially achieve with a more formal structure” (S7).

### Impacts of Follow-on Groups

#### Social Connections and Emotional Support

Participants spoke of the importance of the ongoing support, trust, understanding, and social connections that FGs enabled: “It’s just like another family” (P15). This supported people’s well-being: “Certainly mentally I am in a better place” (P3). Most groups discussed and supported each other through flare-ups: “I think we all have our flare-ups definitely. It’s something that we can all talk about” (P18). In two cases, people spoke of how FGs had supported people in crises:

“I can remember spurting out all this stuff and they said, ‘You need help. You’ve gotta go and see your doctor. Please phone your doctor now. Make an appointment. Go and see them. You need help!’ One of the members of the group said, ‘I will physically take you there’” (P12).

Another situation arose on a WhatsApp group, where a person posted a very worrying comment and a group member alerted a clinician:

“What happened is one of the group members alerted us … the patient who was deemed to be at risk was contacted by a member of staff here” (S5).

One interviewee spoke of how such a situation was deeply concerning:

“One [person] in particular would say ‘I’m having a really bad day. This is awful, I can’t carry on.’ And then everyone would respond ‘Oh no! What’s wrong? What can we do? Come on, this is gonna be okay.’ And then she would go silent … we might not hear from her for a couple of days despite us all texting her …. So that was really stressful … has she done anything silly?” (P25).

These difficult situations appeared to have different impacts on the groups. A group that regularly met face to face and had strong bonds, seemed to strengthen their connections as a result. In contrast, the WhatsApp group later disbanded, and people just kept one-to-one friendships where they chose to.

Other risks of FGs were explored through interviews. One staff member expressed concerns of the possibility of FGs turning into a “moaning shop” (S4). Patients who attended FGs were aware of this potential and several highlighted the importance of having “fun” (P3, P7, P11, PTV4, PTV6, PTV10) and friendship and enjoyment (P3, P4, P7, P8, P9, P10, P14, PTV7, PTV8, PTV10). There was one incident spoken of where disagreements had occurred within a FG which members found difficult but was resolved together. No interviewees spoke of negatively comparing themselves with others, although three (P2, P6, P18) shared how they were sad that their pain or ill-health meant that they were less able to attend groups than they would have liked to.

#### Self-Management Skills in Everyday Life

FGs provided a space to share and try out different self-management techniques that had been learnt on the PMP:

“Different people take and gain different things from it …. Some people have really got into the physical side of channeling themselves through things, whereas others have got more interest in doing a set period of relaxation and some do set exercises at a set time of day, and so it’s just taking what you’ve learned from the program really and working out what’s useful for you and what isn’t that useful and then having the motivation to actually do it” (P1).

“What’s useful is if I’m having a bad day or [name of P8] or [name of P14] whatever, one of us will remind the other to, you know, slow down and we’ll remind each other of the techniques we’ve learnt” (P10).

Five people spoke of changes in their relationships with medication and health professionals, or less need to access health services:

“I’ve halved my medication since the group …. That came about with the [follow-on] group” (P11).

“I don’t ever go to my GP for my pain now. Never …. Actually the people that are living with pain are far better to talk to [laughs] than the GP” (PTV5).

Four people spoke of increasing their levels of physical activity. Most groups included conversations about information sharing and symptom management which could improve people’s ability to cope. Five FG participants (P3, P9, P11, P12, P15) said groups helped with acceptance and confidence and supported them to feel in charge of their lives: “It made me get in control of the illness and of my life more” (P9). While connections between people were important in initially forming FGs, in the groups that had been going for the longest periods of time there was also evidence of a “dose effect,” where close friendships were strengthened, with greater impacts.

“As time’s gone on … it’s gone from a tap on the shoulder to a little hug and now … you can see the love. There’s definite love between people now and I just think that’s wonderful. And I could never have believed that when we first started, you know. I just thought we’d have a cup of coffee and a chat and that would be that” (PTV9).

Where group membership dispersed some people’s ability to reinforce their self-management skills, both through the PMP and/or FG, meant their lives moved on, and they no longer felt a need to keep in touch.

## Discussion

This study examined to what extent and how patient-initiated and led peer support groups after PMPs facilitate enhanced well-being, self-efficacy and improved pain self-management skills for their participants.

Our data show that peer support can tackle previously identified barriers to pain self-management [44], including limited support from family and friends; lack of strategies to specifically tailor pain management techniques to people’s daily lives; difficulties in maintaining strategies after PMP courses; and difficult patient-physician interactions. People could develop strong cooperative relationships within peer support FGs; this reciprocity could mean there was less of a feeling of isolation or reliance on family or friends who may not understand chronic pain. Group members applied self-management techniques within their everyday activities, and some interviewees discussed how peer support had changed their relationships with clinicians, alongside reducing use of healthcare services. Our findings align with a study that analyzed one-to-one peer support for ex-members of the armed forces with chronic pain [8] and builds further evidence to show that peer support participants value interpersonal connections, the reciprocal nature of support, and discussions on how to use pain management techniques. Further research is needed to compare long term health outcomes between those PMP participants who attend FGs, with those PMP participants who don’t, alongside patients’ ability to cope with pain, depression, social activity and how much patients use health services.

The distinctiveness of peer support, in comparison with clinical treatment, is that it can be naturally-occurring, in real-world interactions between people [45, 46]. Study data illustrate the value of friendship and “having a laugh” between group members. The importance of humor within pain treatment has been highlighted [47], this study illustrating the importance of natural laughter that comes from friendship and shared experiences. Experiencing long-term pain can challenge some fundamental social and basic human needs, such as the need for autonomy (including issues of dependence and loss of agency), and the need to belong (including feelings of social exclusion and loneliness) [20]. Peer support had a clear impact on participants’ sense of belonging, enhancing social activities, enabling more social connections, increasing personal agency and reducing isolation. They provided an autonomous space for reciprocal support of fellow members, where people did not need to explain the impact of living with pain and could support each other to gain a sense of control over their lives.

This study contributes to other aspects of PMPs that are currently underreported, including the “active ingredients” of change from patients’ perspectives and how social relations between PMP patients can affect PMPs and their outcomes [48, 49]. PMP participants can react differently to information and emotional support from clinicians (sourced from professional training) and people who have experienced pain (stemming from contextual, lived experience) [50]. In the PMPs, patient tutor volunteers could act as inspiring role models, where their “experiential authority” [51] was illustrated through the embodiment of self-management capacities and techniques, which could alter people’s understandings and behaviors of what was possible when living with pain. Whilst theories of social learning [52] have had considerable influence on PMP development, further thinking about how positive social modelling can be used to support learning and elevate coping mechanisms beyond PMPs may be helpful. Study data illustrate that intentionally promoting the social connections made between participants within a group PMP, through the encouragement of sharing contact details and follow-on meetings can enable longer term social support amongst PMP participants, after a PMP has finished. The social connections, relationships and understanding of each other’s experiences within the PMP and beyond within the peer support FGs were a key source of learning. PMP facilitators need to enable these interactions [26], so that people’s own experiential knowledge of living with pain can enable groups to be relevant and mutually beneficial [50]. Fostering PMP participant connections can then support longer-term peer support between participants after PMPs have finished. However, peer support groups did not always form in a way that included all PMP members. Some people positively opted out, but other issues of social exclusion may be operating and health professionals' awareness of interpersonal dynamics amongst group members can help prompt inclusion as group members make their decisions about forming their peer support group.

This peer support social intervention comes from a “real-life” problem that patients identified at the end of their PMP. The peer support strategy derived from PMP patients ideas and was co-produced by them with clinicians’ support, in the context of their own lives. Study data illustrate how peer support groups follow core principles of co-production [13], fostering peer support networks and reciprocal relationships, enabling people to support each other’s capabilities and capacities, and providing a space to exchange both professional and experiential knowledge. Further work is needed within pain services to embed “the expertise of the person with chronic pain” [10] to improve healthcare quality [53].

### Clinical Implications

Clinicians need to address the social implications of pain within their work, as supporting the interpersonal needs of people with pain is associated with physical and psychological well-being [20]. This research highlights the importance of group-based peer support to facilitate social connections and share lived experiences through mutual, reciprocal relationships. While peer support has been evidenced to have potential, clinicians have said that time and space are the biggest barriers to implementation [1]. This peer support intervention requires relatively little time commitment from clinicians because patients are asked if they want to take the idea on and organize this, with some minor adjustments to the structure of the end sessions of PMPs. The original peer support protocol assumed that patient tutor volunteers who co-facilitated the PMPs were important to the development of groups, however our data suggests that where strong bonds were developed between PMP participants, the need for patient tutors to support the set-up of groups diminished. The authors have developed implementation materials to support the establishment of peer support groups [41–43] (Supplementary file 2, Supplementary file 3). While there have been many positive aspects of peer support illustrated, clinicians need to consider potential negative consequences when setting these up. These include potential risk around members supporting others at times of severe need or crises (where members may need to reach out to professional support). The NBT pain service in this study maintains open access consultation-support for members if such situations arise. In addition, relationships may not always be positive, so it is important to encourage individuals to assess whether a peer support group is an appropriate support mechanism for themselves.

Further developments of the peer support group intervention include connecting different groups within particular geographical areas so that groups can join if they wish and ensuring equitable access. This may also be a time limited process as groups can fade as time elapses after a PMP, although we also found a couple of examples of where groups had been meeting for over five years.

This research was completed and implementation materials launched [41, 42] (Supplementary file 2, Supplementary file 3), including a short film about follow-on groups [43] shortly before the COVID-19 pandemic, which has drastically affected the way that face-to-face PMPs can be delivered, with recommendations to suspend in-person meetings during the pandemic [54]. Several studies have highlighted the potential of delivering pain self-management interventions in a remote format including online [55–57]. However caution is needed, with potential issues in relation to access and engagement, and the challenges of creating “therapeutic alliance” remotely [58]. Studies that are addressing how to manage chronic pain during the pandemic [54, 58] have yet to include the voices of people with chronic pain [59]. Online peer support has been advocated during the COVID-19 pandemic [60]. There are several studies that highlight the potential for peer support mediated through the internet, with promising evidence that internet-based peer support can lead to improvements in pain intensity, health distress and self-efficacy [61]. Within this study there were different examples of peer support groups using the phone, email and social media to connect with each other, however more research is needed to understand the experiences and efficacy of this form of connection.

Study data come from a “real-life” intervention, co-produced by PMP patients within their own everyday environments. It fills a research gap to examine how group peer support can extend beyond professionally facilitated programs, exploring how peer support can facilitate shared learning, emotional support and the development of agency [26]. In focusing on both attenders and non-attenders, both positive and difficult experiences of PMPs and peer support groups are analyzed to understand the less explored challenging experiences of group self-management programs [26]. A limitation of the study is that the majority of PMP participants interviewed were white British, which should be taken into consideration when interpreting results. While local NHS monitoring of PMP referrals has previously shown that ethnicity roughly matches the local population, it is unclear if this is also true for those who choose to join an FG or participate in research.

## Conclusion

This study illustrates how patients can have a vital co-production role in identifying a service gap, designing, implementing and refining a solution to meet their needs for peer support that follows on from PMPs. Results indicate that this ongoing peer support engages some people with chronic pain as they complete professional-led treatment, providing emotional, practical and social benefits to those who attend. Peer support groups can maintain benefits gained from the PMP and can reduce reliance on medication and health services. They may support a phase of adaptation to chronic pain that is not sufficiently covered during a PMP course, which is longer term social recovery, the third element of the bio-psycho-social model of care. However, peer support groups are not without their challenges and may need ongoing clinician support if a group member experiences an acute crisis.

## Author Contributions

J.H., M.F., H.B., R.P., M.J.L., N.A., S.V., H.W., and S.W. all made substantial contribution to study design. N.A., S.W., and S.V. were responsible for study conception. J.H. and N.A. were responsible for study management and coordination. S.V. contacted and enabled interview recruitment. M.F. and H.B. interviewed participants, observed FGs and analyzed the data; J.H., R.P., H.B., M.F., M.J.L., N.A., S.V., H.W., and S.W. discussed results. M.F. drafted the paper. All authors read, commented on, and approved the final manuscript.

## Supplementary Material

pnab189_Supplementary_Data

## Notes

Funding sources: This research was funded by the National Institute for Health Research Applied Research Collaboration West (NIHR ARC West). The views expressed in this article are those of the authors and not necessarily those of the NIHR or the Department of Health and Social Care.
