# Supplementary material for: Experiences of Patient-Led Chronic Pain Peer Support Groups After Pain Management Programs: A Qualitative Study
Source: Pain Med. 2021 Jun 28;22(12):2884–95. doi: 10.1093/pm/pnab189 (PMC8665998; doi:10.1093/pm/pnab189)
Supplement: pnab189_Supplementary_Data [file pnab189_supplementary_data.zip › pnab189-suppl_data/Supplementary file 1 Interview questions clean.docx]

# Follow-on after pain management programmes - Interview questions

# Clinical staff interview questions

Personal role –

1. Can you tell me a bit about your job, how long you’ve been working in this role? Can you explain your role in the (PMP/SMP)? How does this relate to the peer-support groups?

### Knowledge and understanding of Peer-support programme –

1. What do you see as the purpose of the peer support programme?
2. How are the peer support groups described and explained in the PMP/SMP and at what PMP/ SMP stages does this happen? (**Think** **transition/bridging**)
   1. How do you support and facilitate the process of the SMP/PMP finishing, and the follow-on groups developing? (**Think self-ownership**)
   2. Is there anything important that you need to do to support this process?
   3. Is there a particular structure to the follow-on groups that they are supposed to adhere to?
3. How do the groups get organised? Does this change over time?
   1. What do you know about the development of the follow-on groups?
   2. Do you think that the groups evolve through time from the original description?
   3. What are the barriers and facilitators of follow-on groups starting and continuing?
   4. Does the number of patients completing the PMP/SMP affect the dynamics of how follow-on groups develop? E.g. if some patients drop out of the SMP/ PMP half way through, does this affect how follow-on groups develop?
4. What are the reporting mechanisms for these groups? How do you know what is happening with all the different possible groups that may be running?

### Opinion of peer support groups –

1. What do you consider the key ways that the peer-support group might help patients? Is this based on any model? Do you think this differs from what patients might want out of the follow-on group? (e.g. Advice, support, social networks?)
   1. What are the key ingredients needed for a peer support group to be run successfully?
   2. Do the different contexts within which they are run affect their success in any ways (e.g. urban/ rural, high/low income areas, need for groups in different languages)?
   3. Are there some people that these groups do not suit? If so why?
2. What do you see as the:
   1. Benefits?
   2. Challenges?
   3. Resource implications?
   4. Any potential risks or negative impacts from peer support groups?
   5. Are there any arrangements for who to contact if follow-on groups are concerned about a member experiencing a serious crisis?
   6. What are the implications in relation to equity for patients who would like to be part of a follow-on group, but don’t have access to one?
3. One thing we’ve noticed is the tension between having an organic evolving process for follow-on groups and the need for active guidance and support. We came across [this model from the Health Foundation](https://qlabessays.health.org.uk/essay/learning-and-insights-on-peer-support/#tensions) that highlights some of these issues. What do you think of this model in relation to follow-on groups?

### Knowledge of patient tutors (Think transition/bridging/change in power balance) –

1. It would be good to understand more about the role of the patient tutors:
   1. What are their main roles? What support do they receive?
   2. Are there particular skills or characteristics that it is useful for a patient tutor to have? (type of person: e.g. altruism, as don’t get paid)
   3. What incentive, if any, is there to become a volunteer?
   4. Are they trained, if so by whom and what are the key components of the training? What skills are important within this training?
   5. What incentives and support do volunteers have for instigating and supporting follow-on groups to develop?
   6. Do volunteers get expenses for travelling to SMPs/ Follow-on groups? If so, what are the arrangements for them claiming this back?
   7. Do volunteers get supervision? If so, by whom, how often?
   8. Are there any challenges or negative impacts that the volunteers may face? If so, how do they address these?
   9. Do you think any further support or training is needed for the role?

**Do you want to add anything else?**

# Patient tutor interview questions

### Role as a patient tutor–

1. Tell me a bit about how/why you became involved as a patient tutor (Own experience of chronic pain, SMP, incentives etc).
2. What skills and characteristics do you need to be a patient tutor? Why?
   1. Do you receive any training, guidance, ongoing support? If so, what was most important/useful?
   2. Do you feel you need any further information, guidance, support for your role?
3. Any potential risks / disadvantages / potential negative effects to this role? How could these be reduced, i.e. changes to the training, more support etc? (Personal experience and how addressed?)
4. How many groups have you been/ are you involved with? Are you still in touch with all the groups? Do they all still meet? Why/ why not?
   1. What roles do you take with the group?
   2. Do the different groups that you’re involved with vary? If so how and why?
5. Do you connect with the other groups that are running/volunteers? If so, how?
6. Do you report what happens in the groups to anyone? Other reporting mechanisms for these groups (NBT)?

### Knowledge and understanding of Peer-support programme –

1. Can you tell me why and how the follow-on programme developed?
   1. What is the purpose of the follow-on groups?
2. What do you think patients want out of the follow-on groups? (e.g. Advice, support, social networks?) Do you think this is different from their intended purpose?
3. How do you present the idea of the follow-on groups to the PMP/ SMP groups? Has this changed? (**Think** **transition/bridging**)
4. How do the groups get organised?
   1. Can you talk me through the process of how the follow-on groups develop after the PMP/ SMP?
   2. Is there a particular structure to the follow-on groups that you are supposed to adhere to?
   3. What is your role in this? Does this change over time?
   4. Do you always withdraw from the groups after 3 group sessions? Why? Have there been any issues with this? (i.e. more time or less time needed to support the groups?)
   5. Have you ever been back to a group after the 3 group sessions? If so why?
   6. How do you encourage self-ownership of the groups? Do you think that this has worked with the groups? Are there any difficulties in enabling the groups to self-organise (e.g. communications, does one patient need to lead?)
   7. Do you know if off-shoots from the main groups have developed? (e.g. sub-groups, friendships?) Have people swapped groups, and if so why? How did this work?
   8. Have people who were not on a SMP/PMP ever joined the group? (Family, carers?)
   9. Do you know if people belong to other support groups?
5. Do you know if there any costs in association with the groups? How are these covered?
6. With the groups that you are aware of, how long have they been running and when do they meet (Day, time, for how long and how often?) **(try to map all the groups)**
   1. Do you know of times when follow-on groups have never started? How often, where, when, types of participants, why?
   2. Do you know of times when follow-on groups have established themselves and then folded? How often, where, when, types of participants, why?
7. Do family/carers have a role?

### Opinion of peer-support groups –

1. What are the key ingredients needed for a follow-on group to be run successfully?
2. Do the different contexts within which they are run affect their success in any ways? (e.g. urban/ rural, high/low income areas, need for groups in different languages?)
3. Is there anything else you would like to mention in relation to the follow-on groups and their:
   1. Benefits?
   2. Challenges?
   3. Key ways that the follow-on groups help support participants?
   4. Any potential risks or negative impacts from follow-on groups? (e.g. potential for decline in high functioning patients, negative comparison with others, having to manage difficult support situations with fellow participants?)
   5. Resource implications?

**Do you want to add anything else?**

# Patient participant interview questions (For those who attend the follow-on groups)

### Personal details –

Collect information on:

- Gender
- Age
- Ethnicity
- Area lives in
- Diagnosis
- Time living with symptoms

1. Tell me a bit about how and why you attended the PMP/SMP, when and where was this? (clarify if PMP or SMP i.e. 8 or 12 weeks?)
2. Did you find the PMP/ SMP helpful? How?
   1. Have you sought further professional help since attending PMP?
   2. Patient details (More needed on how their life has been post PMP?)

### Knowledge and experience of attending a peer-support group –

1. How was the idea of a follow-on group presented to you? What did you think initially? (**Think** **transition/bridging**)
2. What follow-on group do you attend? How long have you been attending this group? Details of the group e.g. **(Think/ask - How does each of the following affect the meeting/impact the respondent?)**
   1. How many patients, where do you meet, how often, do people have a variety of conditions or similar? How long have you been meeting?
   2. What is the age range of your group? (i.e. are you similar ages or is there a broad range of ages?) How many men and how many women usually attend your group? (Gender/ age range of different group participants?)
   3. How does the group get organised? How do you decide dates and venues? Has anybody taken the lead; if so, how and why?
   4. How do you meet - face to face, online or via phone? Why? What are the benefits and challenges of this?
   5. What time of day/day of the week does your group meet? What is the average length of a meeting? Why? Do some people work full-time?
   6. How do you communicate with each other to arrange meetings? (Facebook, texts, emails, phone calls?)
   7. Have many patients dropped out of the group? Do you know why?
   8. Does this suit you or would you prefer to meet differently i.e. day, place/medium etc.?
3. Can you talk me through what happens in a typical group meeting and how is this decided? **(think/ask how these factors might have an impact)**
   1. What do you talk about? Do you discuss your pain?
   2. Do you discuss things from the pain management programme? (e.g. the mutual support plan that was developed, goal-setting etc.?)
   3. Do you discuss other pain management methods?
   4. Have you done exercise together as a group?
   5. Do you feel comfortable in the group? Are you able to share freely?
   6. Has anything happened in the group that you have been uncomfortable about? Can you explain why?
4. Do you have a particular role? Do some people take more active roles in the group than others? How does this work in practice?
5. Do decisions get made within the group and if so how and about what?
6. Are there any costs associated with attending/running the groups? (i.e. travel, venue, refreshments etc.?) How do these get covered?
7. Is there any more support that you need to function as a group?
8. What do you want to get out of the follow-on group? Do you think this is the same as other participants, or do different group members want different things from being in the group? How does this affect the meetings? **(Think social network typologies)**
9. Do you know if off-shoots from the main groups have developed? (e.g. sub-groups, friendships?)
   1. Do you contact people in the group outside of group meetings? How often? Why? Does this help, and if so how?
   2. Have people who’ve not been on the original SMP/ PMP ever joined the group?
   3. Have people swapped groups, and if so why? How did this work?
   4. Do any members family or friends join you? Do they have a role?
10. Do you know of or link with other follow-on groups? Would you like to?
11. Do you receive support for your pain management from any other source – family, friends, clinicians or other support groups etc?

### Opinion of the peer support groups –

1. Why do you attend the groups? Do you attend regularly – and why is that?
   1. What are the benefits that you get out of attending them? (impacts on self-efficacy, connection with others, social/emotional/practical support, information?)
   2. What is it about the group that makes a difference to you?
   3. How long do you think you will continue attending?
   4. Are there any challenges in running or attending the groups?
   5. Is there anything you would like to change or do differently in your group meetings? Why?
2. Has attending the group made any difference to how you self-manage your condition? How?
3. Have you experienced any flare-ups or crises since attending the follow-on groups?
4. If so, who did you get support from during this crisis/ flare-up? Did you share this with the group and if so did group help in any way to manage your symptoms/avoid the need for further treatment? Did you need to access medical support, and if so how?
5. Without mentioning names, have other people in the group experienced flare-ups or crises? How did the group support this person?
6. What are the key ingredients needed for a follow-on group to be run successfully? (prompt for any context issues e.g. geographic area)
7. Do you think that there are any risks or adverse effects in attending the groups? (E.g. potential for decline in high functioning patients/dependency, negative comparison with others, having to manage difficult support situations with fellow participants etc?)
8. Does your pain affect your ability to do every day activities e.g. work, socialising, hobbies etc? How? (Something about confidence) Does being a member of the follow-on group help or hinder in any way?

### Patient tutor role -

1. What do you see as the role of patient tutors?
2. Do you remember how the patient tutor supported the development of your group? Is more or less support needed for the group and if so why? (**Think** **transition/bridging/power** **shifting**)

**Do you want to add anything else?**

# Patient participant interview questions (For those who have opted not to attend/dropped out of the groups)

### Personal details –

1. Tell me a bit about how and why you attended the PMP/SMP, when and where was this? (clarify if PMP or SMP i.e. 8 or 12 weeks?)
2. Did you find the PMP/ SMP helpful? How?
   1. Was a patient tutor involved in your SMP/PMP? If so, what do you see as their role? Was it helpful to have their perspective in addition to the clinicians?
3. How did you feel after the PMP/SMP about managing your pain? Has this changed over time?

Can you give me a bit more detail on your background before you went to the PMP/SMP? Patient details (gender, age group, ethnicity, geographic area lives in, diagnosis, how long they have been living with the condition?)

### Managing after the SMP/PMP –

1. Do you still use some of the skills/activities you learnt at the PMP/SMP?
   1. i.e., goal setting, problem solving, stress management, sleep, positive/helpful self-talk, pacing and setting baselines.
   2. Do you use the relaxation exercises?
   3. Have you experienced any flare-ups or crises since attending the SMP/ PMP? If so, who did you get support from during this crisis/ flare-up? Did you need to access medical support, and if so how? Have you sought other professional help since attending SMP/PMP?
   4. Do you still use the activity monitor diaries?
   5. Are you comfortable with communicating with people and healthcare professionals about your pain?
2. Were you provided with any guidance following on from the PMP/SMP?
   1. If so what was useful?
   2. If not what would have been useful?
3. Does your pain affect your ability to do every day things e.g. work, socialising, hobbies etc? How? (Something about confidence)
   1. Do you get any support with this?

### Knowledge and/or experience of attending a peer-support group –

1. Did you have the option to join a follow-on group?
   1. How was the idea of a follow-on group presented to you? What did you think initially? Do you know if your group ever met up?
   2. Could you choose to join at a later date if you wanted to?
   3. Did you ever attend a group? If so what lead to your stopping? If not why? (Possible prompts)
      1. Time
      2. Locality
      3. Costs
      4. Not helpful, don’t like groups or didn’t want to spend more time with the others in the group
      5. Medium of meeting, e.g online etc.
      6. The group folded/ did not get off the ground
2. What might have made you make a different choice?
3. Do you belong to any other support groups? **(Think social network typologies)**
4. Do you receive support from any other source? (Think of family, friends, clinicians etc?)
5. Are there any other types of further support that would be useful to you?
6. Is there anything else that you have found helpful to manage your pain?

**Do you want to add anything else?**
