# Supplementary material for: Experiences of Patient-Led Chronic Pain Peer Support Groups After Pain Management Programs: A Qualitative Study
Source: Pain Med. 2021 Jun 28;22(12):2884–95. doi: 10.1093/pm/pnab189 (PMC8665998; doi:10.1093/pm/pnab189)
Supplement: pnab189_Supplementary_Data [file pnab189_supplementary_data.zip › pnab189-suppl_data/Supplementary file 2 Facilitators follow-on leaflet.docx]

Follow on
groups after pain management programmes

Watch people talk about their own experiences of follow-on groups:
[bit.ly/Peer
SupportGroups](http://bit.ly/PeerSupportGroups)

Guidance for Pain Management Programme (PMP) facilitators in setting up follow-on groups

A follow-on group is an informal group, where people who have completed a PMP continue to meet and catch up, but without NHS pain management staff. Research has shown that follow-on groups can help PMP participants support each other to manage their pain, improve well-being and connect socially in the longer term.

This is guidance for PMP facilitators, to enable the development of follow-on groups. This guidance accompanies the patient information leaflet that is to be given to PMP participants, which covers the personal benefits and organisation of follow-on groups.

# When to introduce the idea of follow-on groups

The stronger the bonding has been within a pain management programme the easier it will be to form a follow-on group. Whilst it is dependent on group dynamics, it is helpful to introduce the idea of follow-on groups three weeks before the end of the programme (e.g. week 6 within an 8-week course). This gives enough time for the group to discuss and agree the practicalities so that they can arrange a date to meet independently by the time the programme finishes.

# What facilitators need to do:

## 3rd session before the PMP end

- Introduce the idea of setting up a follow-on group and give PMP participants the
  follow-on group leaflet for patients. Give people time to discuss this.
- Explain that it can be helpful for people to meet up, and share how life has been going for them, after the PMP is finished. The group is not all about talking about pain, but for help and support with people you trust.
- Show the video of patients describing the follow-on groups, hosted by YouTube and available at: [bit.ly/PeerSupportGroups](http://bit.ly/PeerSupportGroups)
- If you don’t have access to YouTube in your PMP, point out the link to patients in their information leaflet and say that they can view the video through this link.
- Say that people can think about it over the next week and decide whether they would like to set up a group.
- This introductory discussion could take about 15 minutes within a session.

## 2nd session before the PMP end

If some people want to start a follow-on group, the group will need some time to organise the following. This could be introduced just before a break so that people discuss these things in the break time. However, ensure that people who go outside or are not in the room are still included. It will be helpful to talk with the whole group after the break to see where they have got to in arranging what they are going to do. Ensure you give the group space to discuss the items below.

### Exchange contact details:

- If people would like to connect, they’ll need to exchange contact details. Explain that not everyone may want to take part, and people don’t have to share contact details if they don’t want to.
- Highlight that it’s good to give at least **two** forms of contact e.g. phone number and email address. Ask people to write clearly, because people have previously lost contact because of mistaken contact details.
- One way to share contact details is to put everybody’s contact details on an A4 sheet of paper. Take this sheet and photocopy it for everyone, so that each person has a copy. Some people may prefer to take a photo of the sheet with their phone.
- Ask people to test out the contact details on each other before the next session, so that people have the accurate numbers and email addresses.

### Agree a time to meet:

- It can be difficult to find a time that suits everyone. The group may need to go with the majority who can make it.
- Not everybody has to go to every meeting. It may help to make it flexible so everyone can make it at some point.
- It may be that two separate groups develop. If so, each group can decide when and where to meet independently of the other.

### Discuss venues:

Ask people to come to the final session with specific examples of where they could meet so these can be discussed and the best option agreed. You might want to get people to think about the pros and cons of different venues.

Things to consider include:

- **Accessibility:** ease of travel, availability of parking, public transport links, disability or wheelchair access, needs of individuals in the group.
- **Local cafes** are easy places to meet up, but affordability needs to be taken into account. Ensure the group is aware they may have less privacy to discuss more sensitive issues in a cafe.
- **Pubs and restaurants:** some groups meet over a lunch or choose pubs where they serve tea and coffee. Think about affordability. A pub may not suit everyone, as alcohol is served. People may have less privacy to discuss more sensitive issues.
- **Privately hired spaces:** Groups have met in community and youth centres where rooms are available. The private space gives people room to share more freely. Groups will need to think about whether there is a way to make hot drinks and refreshments. If people have to pay for the space, they’ll need to agree how to share the costs. Splitting the costs in advance can avoid some people being out of pocket, if others don’t turn up to the meeting.

### Communicating with each other:

- Ask the group how they plan to communicate with each other. This could include email, phone, text, WhatsApp, Facebook. Highlight that not everyone
  may use social media, so a range of methods may be needed. Make sure that everyone gets all the information, even if they use a different contact method.
- It may be helpful to set up a system so that groups are not waiting for others to arrive that are not coming to a meeting e.g. texting the group so people know who’s coming or not.

# Last session of the PMP

- **Contact details confirmed:** Make sure that everyone has tested out each other’s contact details and everyone has these correctly noted.
- **Meeting agreed:** Give the group space and time to finalise when and where to meet. Advise that it can be good to meet soon after the PMP finishes to keep up momentum. By the end of the session, the group should have a date, time and venue in their diaries before they leave. If they don’t have this date, there is less chance that they will meet up.
- **Emergency details explained:** Over the
  last seven years these groups have been operating, there have been some rare occasions where a group participant has needed support in a crisis. There are several ways that people can get medical and emotional support at these times.
  Show where these numbers are on the patient leaflet.
- Please also give a pain management contact at your local NHS Trust if this is possible e.g. include this under the heading ‘Local pain management services’. The patient leaflet is editable in Word for this purpose.

## Supporting follow-on groups

You may want to keep a connection with follow-on groups to find out how things have been going for them. At North Bristol NHS Trust, where these groups first developed, the PMP team have set up get-togethers for anyone involved in a follow-on group. These usually happen twice a year and are a chance to discuss how things are going, exchange what has been learned, and make contacts with others. You may want to think about setting up something similar in your own area.

## Evaluating follow-on groups

To find out how follow-on groups are developing, we have created a follow-up questionnaire that can be given to patients to find out if they have attended a follow-on group, and their experiences of this. You may want to adapt this questionnaire to ask extra questions that you are interested in. This can be given to patients between 3-6 months after their PMP ends, depending on when you may collect other PMP evaluation data. If you already collect Patient Reported Outcome Measures (PROMs), you could extend the time that you collect these, to find out about longer-term outcomes, differentiating between those who attend follow-on groups and those who don’t. Your measures may already include, or you might want to add:

- Pain self-efficacy questionnaire (PSEQ)
- Patient Activation Measure (PAM)
- Hospital Anxiety and Depression
  Scale (HADS)
- Social Provisions Scale (SPS)
- EuroQol 5-Dimensions 5-Levels
  (EQ-5D-5L)
- Self-report of number of pain relapses
- Resource use questionnaire - GP visits, hospital admissions, pain clinic attendance, medication use.

# About this leaflet

Patients from North Bristol NHS Trust who wanted more support after finishing their pain management course, first set up follow-on groups. They have been introduced at the
end of every North Bristol NHS Trust PMP since 2012.

This leaflet was produced by patients, patient volunteers and clinicians from North Bristol NHS Trust and researchers from NIHR ARC West (National Institute for Health Research Applied Research Collaboration West). They worked together to investigate people's experiences and the effectiveness of
follow-on groups.


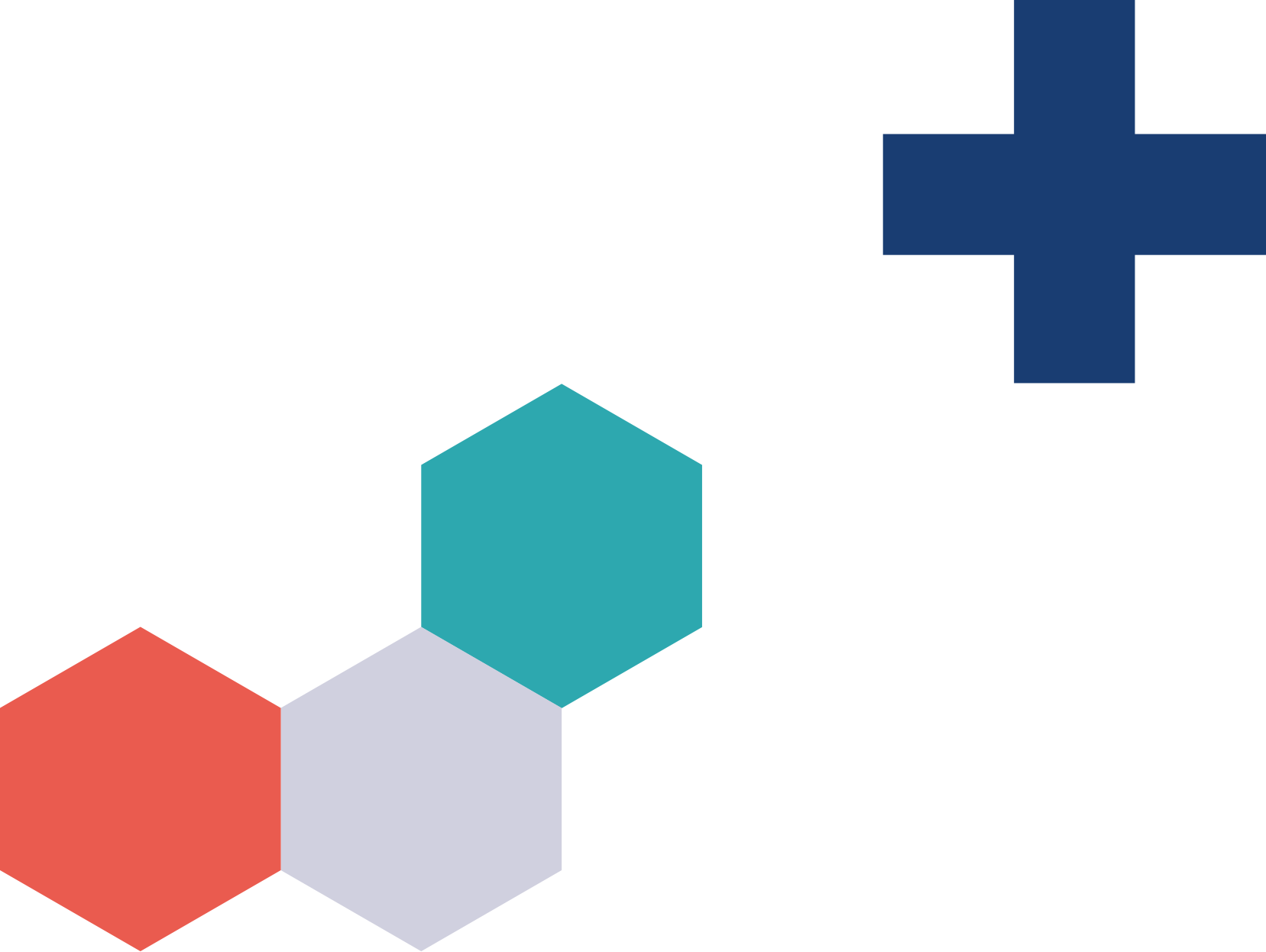


Watch people talk about their own experiences of follow-on groups:
[bit.ly/Peer
SupportGroups](http://bit.ly/PeerSupportGroups)

| This research was funded by the NIHR Applied Research Collaboration West (NIHR ARC West), at University Hospitals Bristol NHS Foundation Trust. The views expressed are those of the authors and not necessarily those of the NIHR or the  Department of Health and Social Care. |
| --- |
| 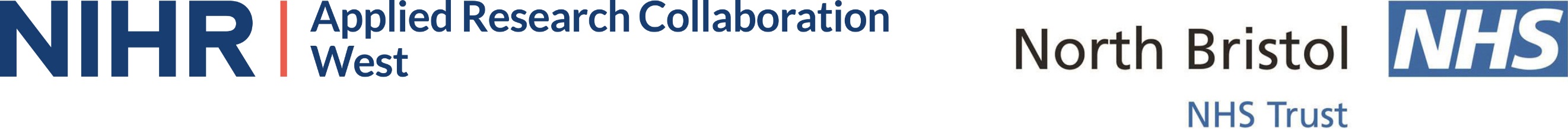 |
