# Supplementary material for: Experiences of Patient-Led Chronic Pain Peer Support Groups After Pain Management Programs: A Qualitative Study
Source: Pain Med. 2021 Jun 28;22(12):2884–95. doi: 10.1093/pm/pnab189 (PMC8665998; doi:10.1093/pm/pnab189)
Supplement: pnab189_Supplementary_Data [file pnab189_supplementary_data.zip › pnab189-suppl_data/Supplementary file 3 Patient follow-on leaflet.docx]

Follow on
groups after pain management programmes: Information for patients

Watch people talk about their own experiences of follow-on groups:
[bit.ly/Peer
SupportGroups](http://bit.ly/PeerSupportGroups)

Why have follow-on groups? Staying in contact with people from your pain management programme (PMP) can be helpful.

A follow-on group is an informal group, where people who have completed a PMP continue to meet and catch up, but without NHS pain management staff. It’s a good way to support each other and share how the things you’ve learnt can help to manage pain and improve your wellbeing. People who’ve taken part in follow-on groups said:

“I don’t have to explain myself. They already understand”

“There’s no judgement from any of the group”

“Even though I feel that
I am managing quite well sometimes, it’s quite nice to just talk to someone who’s going through the same thing as you as well”

# Benefits of follow-on groups

- “We give each other ideas of like
  ‘this works for me.’”
- “We are friends and have a good laugh.”
- “It has been my lifeline.”
- “People will just say to me, ‘Pacing.’
  And I think, ‘Oh gosh. Yeah, I haven’t
  been doing that at all.’”

# Ideas for follow-on group activities

- meet up for a social catch up in a cafe
- go on outings and visit places of
  interest together
- go out for a meal
- go for short walks
- share skills such as knitting, painting,
  arts and crafts.

The groups aren’t only for talking about pain. It’s a great chance to catch up with people socially. It can be helpful to discuss how
you’re using the things you’ve learnt in the PMP, for example:

- pacing
- sleep routines
- how to manage flare ups
- exercise
- goal setting

# Arranging to meet up

If you would like to start a follow-on group
and meet up, you’ll need to:

1. **Exchange contact details:**
   - It’s helpful to give two forms of contact to each other e.g. phone number and email address.
   - Write them out clearly
   - Test them before the PMP finishes,
     so that you know you’ve got the
     right details.
2. **Decide a time:** sometimes it can be hard to find a time that suits everyone. You may need to meet when most people are free. Also, you could set up two groups if people can do different times.
3. **Agree a venue:**
   - How easy is it to travel to,
     near bus routes?
   - Is there parking?
   - Make sure that it is accessible.
     Some people may be less steady on
     their feet or need wheelchair access.
   - Refreshment facilities: is there a place
     to get or make drinks?
   - Be aware of the cost of wherever you choose to meet.

**Options include:**

- - Cafes
  - Restaurants or pubs
  - Community spaces such as community halls, library rooms (there may be a small charge to hire a room/space)

Public spaces like cafes may have less privacy to discuss more sensitive issues or concerns.

| 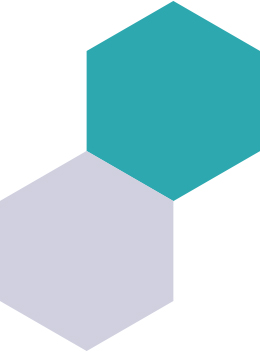 | “If people have said they’ve been struggling, then we know what to suggest – have you tried this or that?” |
| --- | --- |

# Keeping in touch

- It’s good to have a date in the diary to
  meet after you finish your PMP to keep things going.
- Discuss the best way of keeping in contact with everyone. It could be email, phone, text, Facebook, WhatsApp. Be aware that not everyone uses social media.
- Discuss how often you want to meet.
- Not everyone has to go to every meeting.
  It may help to make it flexible so everyone can get to the group at some point.
- It may be helpful to set up a system so that groups are not waiting for others to arrive that are not coming to a meeting e.g. text the group if you’re not going to a meeting.
- It’s good to let someone know if you don’t want to be part of the group anymore.

# What if the follow-on group is not right for you?

If you’re not happy about the way the
follow-on group is going for you, you may want to speak to someone in the group that you trust about this. If you can’t talk to anyone in the group, you might need to talk to your local pain management services. You can decide to stop going to a group at any time if it’s not working for you.

# Worried about someone’s safety in the follow-on group?

If someone is very unwell, there are different ways that people can get medical and emotional support at these times. You could help someone to attend:

- an emergency appointment at their GP
- a NHS walk-in centre or urgent care centre
- in a genuine life-threatening emergency, your local Accident and Emergency department.

Other group members may also need support at this time, so it is important for members of the group to access any services or advice that they need as well.

# Other support available:

## Local pain management services

You can contact your local pain management services using the contact details on the letter you got when you joined the pain management programme.

## Pain Concern Helpline: 0300 123 0789

Pain Concern is a charity working to support and inform people with pain and those who care for them. This is a free helpline service. You can call without giving your name and they will keep anything you tell them private. Every Monday, Thursday and Friday
10am-12pm and 2pm-4pm. Website: [painconcern.org.uk](http://www.painconcern.org.uk)

## Versus Arthritis: 0800 5200 520

A charity that provides information on more than arthritis including fibromyalgia and ideas to manage chronic pain. Free helpline open Monday–Friday, 9am–8pm. Website: [versusarthritis.org](http://www.versusarthritis.org)

## Samaritans: 116 123

Whatever you're going through, call Samaritans free any time, from any phone. Free helpline 24 hours 365 days.

## National Citizens Advice: 03444 111 444

Provides advice on any issue, very helpful for benefits and debt advice. Adviceline is available 9am to 5pm, Monday to Friday. It’s usually busiest at the beginning and end of the day. Closed on public holidays. Calls to Adviceline cost the same as calls to landline numbers. If you’d like to talk to someone face to face, you can find your local advice centre on their website: [citizensadvice.org.uk](http://www.citizensadvice.org.uk)

# About this leaflet

Patients from North Bristol NHS Trust who wanted more support after finishing their pain management course, first set up follow-on groups. They have been introduced at the
end of every North Bristol NHS Trust PMP since 2012.

This leaflet was produced by patients, patient volunteers and clinicians from North Bristol NHS Trust and researchers from NIHR ARC West (National Institute for Health Research Applied Research Collaboration West).
They worked together to investigate
people's experiences and the effectiveness
of follow-on groups.

| 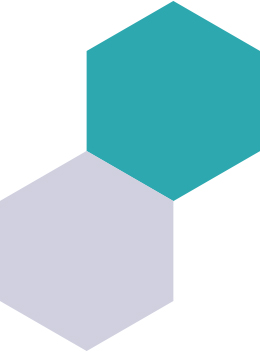 | Not everyone will want to take part, that’s fine. |
| --- | --- |


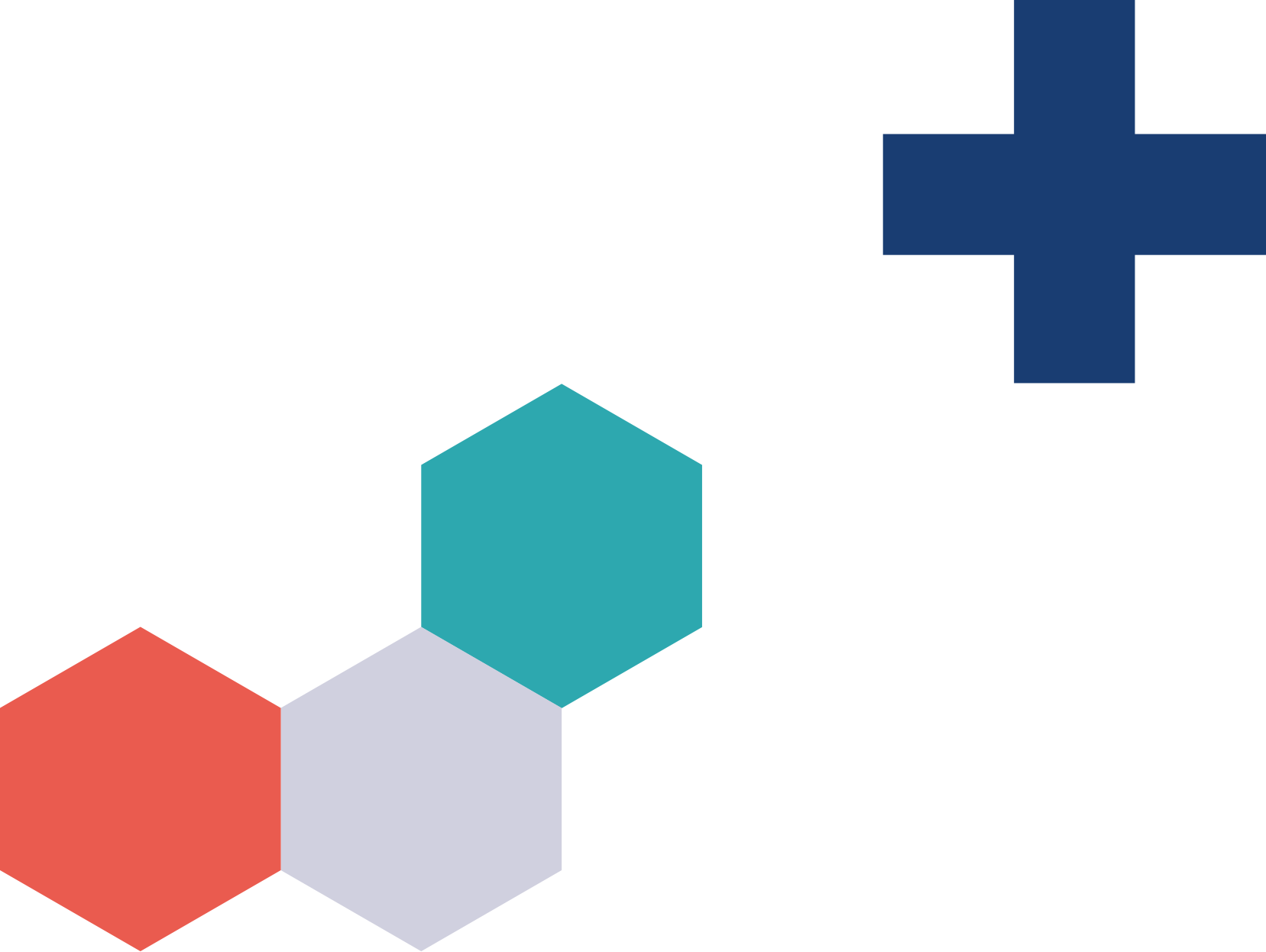


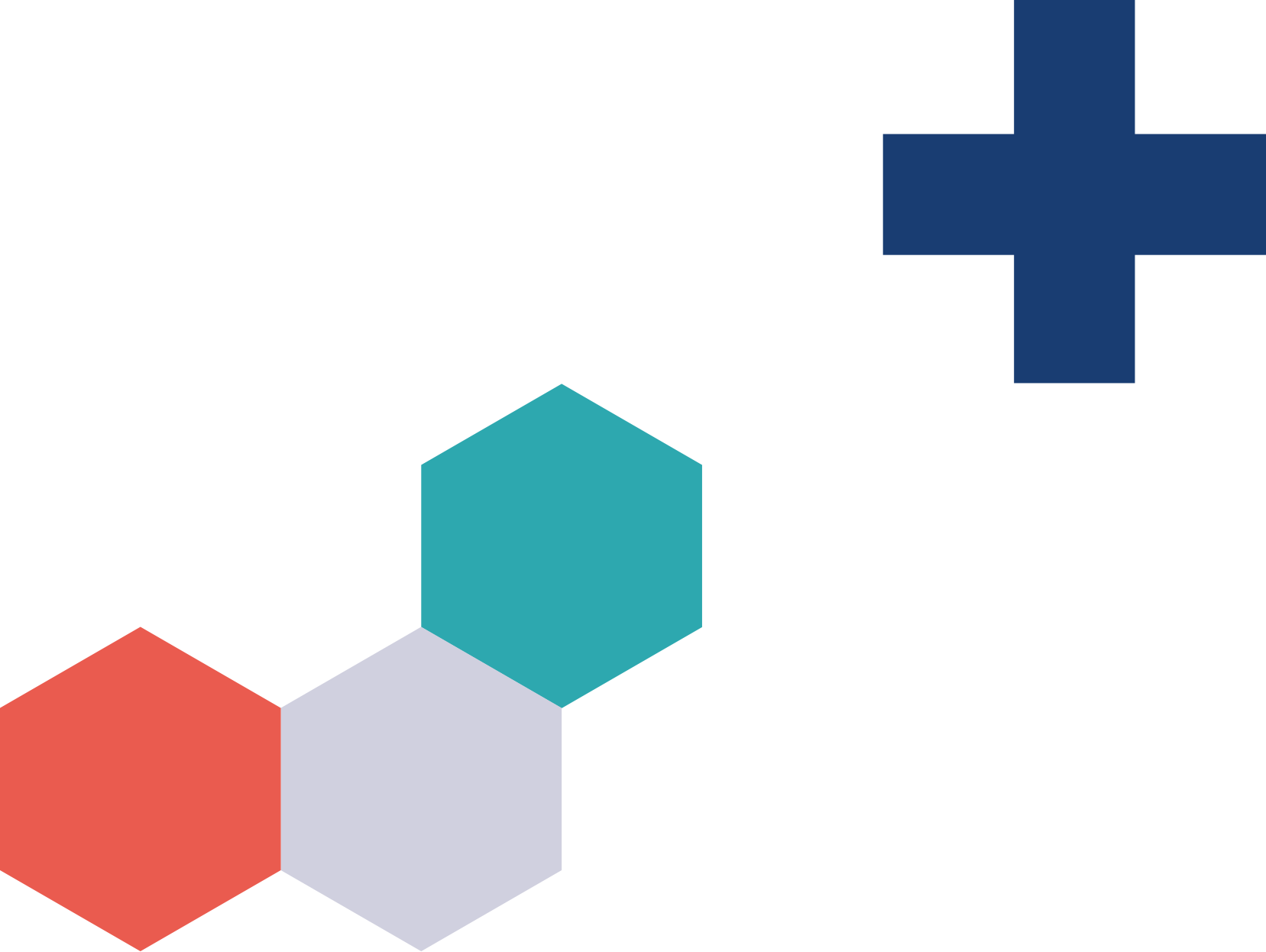


Watch people talk about their own experiences of follow-on groups:
[bit.ly/Peer
SupportGroups](http://bit.ly/PeerSupportGroups)

| This research was funded by the NIHR Applied Research Collaboration West (NIHR ARC West), at University Hospitals Bristol NHS Foundation Trust. The views expressed are those of the authors and not necessarily those of the NIHR or the  Department of Health and Social Care. |
| --- |
| 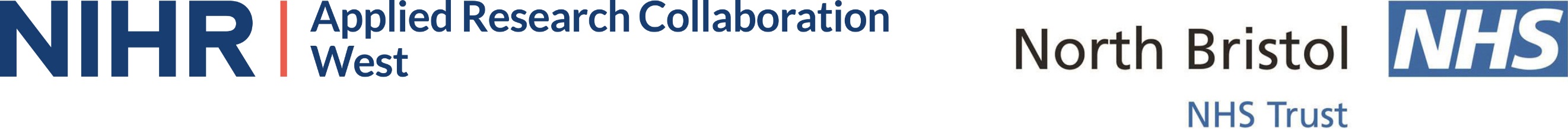 |
